# Concomitant ipsilateral subcapital and intertrochanteric fractures of the femur: a case report

**DOI:** 10.1186/1752-1947-4-363

**Published:** 2010-11-15

**Authors:** George Loupasis, Panagiotis G Ntagiopoulos, Antonios Asimakopoulos

**Affiliations:** 1 2nd Orthopaedic Clinic, General Hospital of Athens 'G Gennimatas', Athens, Greece

## Abstract

**Introduction:**

We report the case of an unusual combination of concomitant subcapital and intertrochanteric fractures of the hip in a patient after a motorcycle accident. To the best of our knowledge, there is no previous report in the literature of these conditions as a result of high energy trauma or of the treatment used.

**Case presentation:**

A 36-year-old Caucasian man was admitted with this rare fracture combination, and was managed with closed fracture reduction and fixation with a dynamic hip screw combined with an anti-rotational cannulated screw. We found four similar cases on reviewing the literature from 1989 to 2009, but these were reports of older patients after low energy injury.

**Conclusion:**

We found that segmental fracture of the femoral neck region is an extremely rare and uncategorized hip injury that can occur not only in older people but also in young non-osteoporotic patients, and should be considered for inclusion in femoral fracture classification systems.

## Introduction

Subcapital femoral fractures secondarily complicating internal fixation of previous united intertrochanteric fractures have been infrequently reported in the literature [1-4]. Simultaneous presentation of ipsilateral neck and intertrochanteric femoral fractures are extremely rare, with only four such cases being reported in the literature during the period 1989 to 2009 [5-8]. All these cases occurred in older patients after a low energy injury.

We report a case of simultaneous ipsilateral subcapital and intertrochanteric fractures in a 36-year old man after a high-energy trauma from a road traffic accident. Based on our clinical experience, the small number of previously published similar cases, the lack of such a fracture pattern in any classification scheme, and a meticulous survey of orthopaedic textbook knowledge, we consider that this is an extremely rare fracture combination, and in terms of patient age, mechanism of injury and the treatment followed, it is to the best of our knowledge, the first such report in the literature.

## Case presentation

A 36-year-old and otherwise healthy Caucasian man of Greek extraction was admitted to the hospital after been involved in a road traffic accident. Our patient was riding his motorcycle at an approximate speed of 70 mph. He collided head-on with a car, which caused him to be thrown from the motorcycle, landing forcefully on his outstretched left leg.

**Figure 1:**
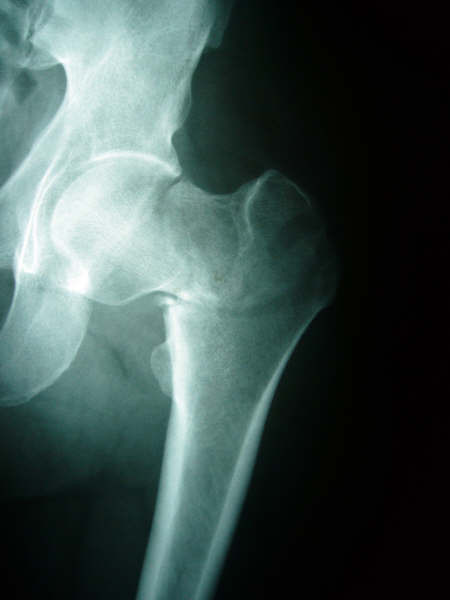
**Pertrochanteric fracture combined with subcapital fracture on preoperative anteroposterior radiograph of the left hip**.

On physical examination, our patient reported pain in his left hip. He was unable to move his left leg, which was shorter than the right and externally rotated. He had no other injuries. Radiographs of the pelvis revealed an undisplaced Garden II subcapital fracture and a displaced intertrochanteric fracture, with the fracture line originating from the greater trochanter laterally, and ending above the lesser trochanter medially. This pertrochanteric fracture could be classified as type 31-A1.1 according to AO/OTA classification (Figure 1)[9].

**Figure 2:**
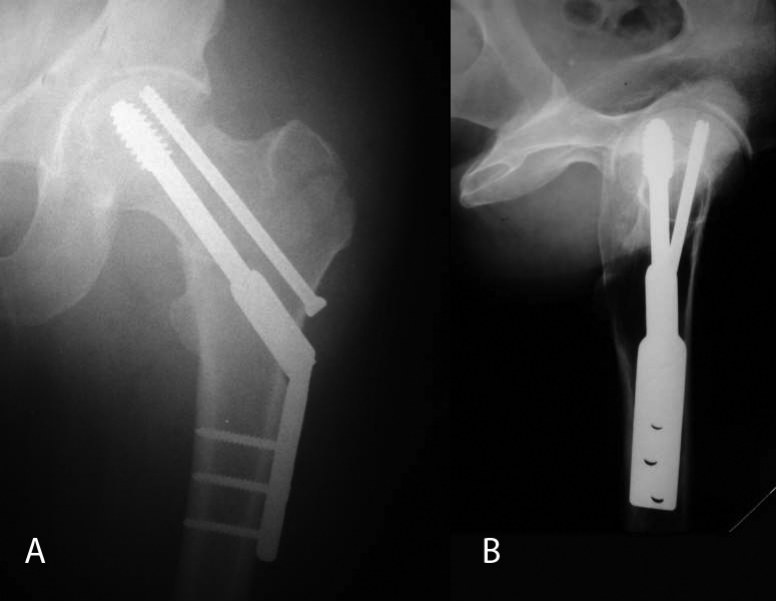
**A) Anteroposterior and B) lateral radiographs of the left hip taken immediately after surgery**.

Our patient was taken to the theatre the same day. Under C-arm fluoroscopy, the intertrochanteric fracture was accurately reduced by closed means, taking care not to apply excessive traction to avoid iatrogenic displacement of the subcapital fracture. The fractures were fixed with a 135° dynamic hip screw (DHS) combined with an anti-rotational cannulated screw (Figure 2). To prevent the development of rotational forces to the femoral head during drilling or lag screw insertion, an eccentric temporary guide wire was inserted into the femoral head and the acetabulum before drilling, and removed after the insertion of the lag screw.

**Figure 3:**
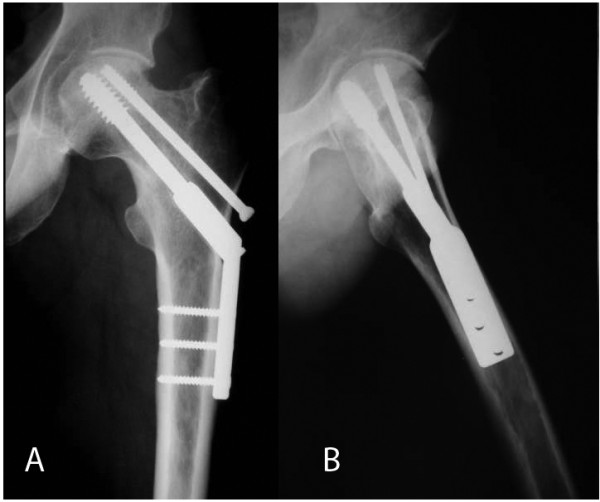
**A) Anteroposterior and B) lateral radiographs of the hip taken two years after the operation**.

Our patient's post-operative rehabilitation protocol included immediate walking with crutches in a touch weight-bearing fashion for eight weeks. Partial to full weight-bearing commenced two months postoperatively. Follow-up included fracture clinic visits at six-month intervals, where the patient was clinically and radiographically assessed and hip functional scores were obtained; Harris hip scores at 12 and 24 months were 88.66 and 93.0, respectively. About one month after the accident, dual energy X-ray absorptiometry was performed at the femoral neck region of the opposite femur, and bone mineral density values were found to be within normal limits. At the last follow-up, two years after the accident, the patient was asymptomatic and had returned to his previous activities, and the fractures were solidly united without evidence of avascular necrosis in the radiographs (Figure 3).

## Discussion

Neck and intertrochanteric fractures of the proximal femur occur predominantly after low-energy trauma in older patients with osteoporosis. The same type of fracture in young patients is usually associated with high-energy trauma.

Although these injuries are one of the most common causes of admission, mainly of older people, to orthopaedic departments, simultaneous occurrence of these fractures on the same side is an extremely rare situation, and only four cases have been reported previously in the literature [5-8]. All four cases concerned older patients who had sustained this unusual combination of fractures after a simple fall. In three of the four cases, the subcapital fracture was either not displaced or only slightly displaced, and the intertrochanteric fracture was displaced but could be classified as a stable injury (AO/OTA 31-A1) [6-8]. All four cases were managed either with closed reduction and osteosynthesis with DHS fixation combined with a cannulated screw, or with DHS fixation alone, with a good final result. In the fourth case, there was a combination of a comminuted intertrochanteric and displaced subcapital fracture, which was treated with a bipolar hemiarthroplasty and cerclage wires with a satisfactory outcome [5].

Our case is of interest because of the occurrence of such unusual combinations of fractures in a younger adult without osteoporosis occurring after high-energy trauma, a contrast to the previously reported cases, which have occurred in patients with osteoporosis. Full preservation of the femoral head should be the main goal of treatment of this complex injury in younger patients. Fixation with DHS supplemented with an anti-rotational cannulated screw should achieve this.

The healing process of the subcapital component of this complex fracture should not be expected to differ from that of an isolated femoral neck fracture. Both fracture lines in our case had an unusual pattern, indicating a different mechanism of injury from that of isolated subcapital or intertrochanteric fracture. Although we can only speculate as to the mechanism of this rare injury, it is likely that the subcapital fracture occurs first when the femoral neck impinges against the acetabulum as a result of an excessive flexion/external rotatory force to the hip. At this stage, if the forces are still not dissipated, a fracture in the intertrochanteric region can occur.

There have been some sporadic reports in the literature about non-concomitant combinations of both fracture types: a primary healed intertrochanteric fracture treated by various methods of fixation, with re-fracturing of the same hip through the femoral neck some time later [1-4]. Several theories have been postulated by various authors regarding the pathogenesis of this fracture pattern, such as inadequate implant fixation[4], imperfect reduction of the primary intertrochanteric fracture[2], and stress protection by the fixation device resulting in local osteoporosis [1,3].

## Conclusion

This report shows that segmental fracture of the femoral neck region is an extremely rare hip injury and can occur not only in the older person but also in younger patients without osteoporosis. Head-preserving and fixation methods should be attempted at all costs despite the combination of two fractures and the great possibility of vascular compromise, and a head-replacing method in a younger patient should be avoided. This unusual complex hip injury has not been previously categorized. Consideration should be given to its inclusion in femoral fracture classification systems.

## Competing interests

The authors declare that they have no competing interests.

## Consent

Written informed consent was obtained from the patient for publication of this case report and accompanying images. A copy of the written consent is available for review by the Editor-in-Chief of this journal.

## Authors' contributions

GL, PGN and AA were the operating surgeons. GL and PGN collected the data and prepared the manuscript. All authors read and approved the final manuscript.
